# An updated review on phytochemistry and molecular targets of *Withania somnifera (L.)* Dunal (Ashwagandha)

**DOI:** 10.3389/fphar.2023.1049334

**Published:** 2023-03-29

**Authors:** Arsalan Bashir, Masarat Nabi, Nahida Tabassum, Suhaib Afzal, Mehrose Ayoub

**Affiliations:** ^1^ Department of Pharmaceutical Sciences, University of Kashmir, Srinagar, Jammu and Kashmir, India; ^2^ Department of Environmental Science, University of Kashmir, Srinagar, Jammu and Kashmir, India

**Keywords:** apoptosis, molecular targets, phytochemistry, withanolides, *Withania somnifera (L.)* Dunal, withaferin A

## Abstract

*Withania somnifera (L.)* Dunal belongs to the nightshade family Solanaceae and is commonly known as Ashwagandha. It is pharmacologically a significant medicinal plant of the Indian sub-continent, used in Ayurvedic and indigenous systems of medicine for more than 3,000 years. It is a rich reservoir of pharmaceutically bioactive constituents known as withanolides (a group of 300 naturally occurring C-28 steroidal lactones with an ergostane-based skeleton). Most of the biological activities of *W. somnifera* have been attributed to two key withanolides, namely, withaferin-A and withanolide-D. In addition, bioactive constituents such as withanosides, sitoindosides, steroidal lactones, and alkaloids are also present with a broad spectrum of therapeutic potential. Several research groups worldwide have discovered various molecular targets of *W. somnifera*, such as inhibiting the activation of nuclear factor kappa-B and promoting apoptosis of cancer cells. It also enhances dopaminergic D2 receptor activity (relief in Parkinson’s disease). The active principles such as sitoindosides VII-X and withaferin-A possess free radical properties. Withanolide-D increases the radio sensitivity of human cancer cells via inhibiting deoxyribonucleic acid (DNA) damage to non-homologous end-joining repair (NHEJ) pathways. Withanolide-V may serve as a potential inhibitor against the main protease (Mpro) of severe acute respiratory syndrome coronavirus 2 (SARS-CoV-2) to combat COVID. The molecular docking studies revealed that the withanolide-A inhibits acetyl-cholinesterase in the brain, which could be a potential drug to treat Alzheimer’s disease. Besides, withanolide-A reduces the expression of the N-methyl-D-aspartate (NMDA) receptor, which is responsible for memory loss in epileptic rats. This review demonstrates that *W. somnifera* is a rich source of withanolides and other bioactive constituents, which can be used as a safe drug for various chronic diseases due to the minimal side effects in various pre-clinical studies. These results are interesting and signify that more clinical trials should be conducted to prove the efficacy and other potential therapeutic effects in human settings.

## Graphical Abstract

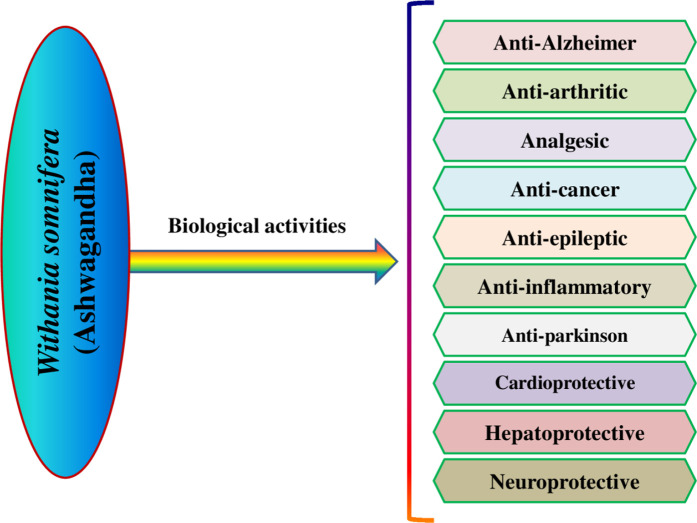

## Introduction

*Withania somnifera (L.)* Dunal is an evergreen woody shrub, commonly known as Ashwagandha (Paul et al., 2021), that belongs to the Solanaceae family, which has 84 genera and over 3,000 species that are widespread across the world (Ghias et al., 2013). It is used extensively as an herbal drug in the Ayurvedic and Unani systems of medicine for the last 3,000 years (Ahmad and Dar, 2017; Behl et al., 2020). It grows abundantly in arid areas stretching from the Mediterranean across tropical Africa, South Africa, and the Canary and Cape Verde Islands, as well as Afghanistan, Baluchistan, Pakistan, Sri Lanka, China, Nepal, and India. It is grown in gardens in warmer parts of Europe and has emerged as a natural weed in South Australia and New South Wales (Paul et al., 2021). In India, it is mostly grown for its fleshy roots, which contain a profusion of phytoconstituents with a multitude of therapeutic values. The plant is widespread in India’s arid areas, notably in Punjab, Gujarat, Uttar Pradesh, Maharashtra, West Bengal, and Rajasthan (Uddin et al., 2012). It has been used in traditional system of medicine as an anti-stress, narcotic, diuretic, combating anaemia, aphrodisiac, etc., for constipation, against worms, liver disease, leprosy, anti-inflammatory, cardiovascular problems, joint pain, antibacterial, nervous system disorders, arthritis, etc. (Behl et al., 2020; Saleem et al., 2020; Paul et al., 2021). Various pharmacological activities have been reported in *W. somnifera*, including anti-inflammatory, analgesic, anti-arthritic, hepatoprotective, anti-cancer, anti-epileptic, anti-Alzheimer, antiparkinson, cardioprotective, neuroprotective, anti-microbial, anti-fungal, anti-oxidant, immunomodulatory, anti-depressant, anti-diabetic, anti-platelet, fibrinolytic, etc. (Ku et al., 2014; Kumar et al., 2015; Paul et al., 2021; Parihar, 2022a). The major bioactive compounds reported in *W. somnifera* are the steroidal lactones called withanolides, the most important ones that are responsible for various bioefficacies are withaferin-A, withanolide-D, and withanone (Saleem et al., 2020; Paul et al., 2021). These bioactive compounds are known to impart the pharmacological effect by targeting different biomolecules in the living systems. They are potent antioxidants and quench free radicals and other reactive oxygen species (ROS) and inhibit free radical induced cell damage. These control the expression of various enzymes, receptors, and other regulatory proteins which are involved in the pathogenesis of various diseases by upregulation and downregulation of transcription factors which controls the production of these regulatory macromolecules (Patel et al., 2013; Saha et al., 2020; Xia et al., 2022). The present review aims to highlight the phytochemistry and molecular targets of *W. somnifera*.

### Phytochemistry

**FIGURE 1:**
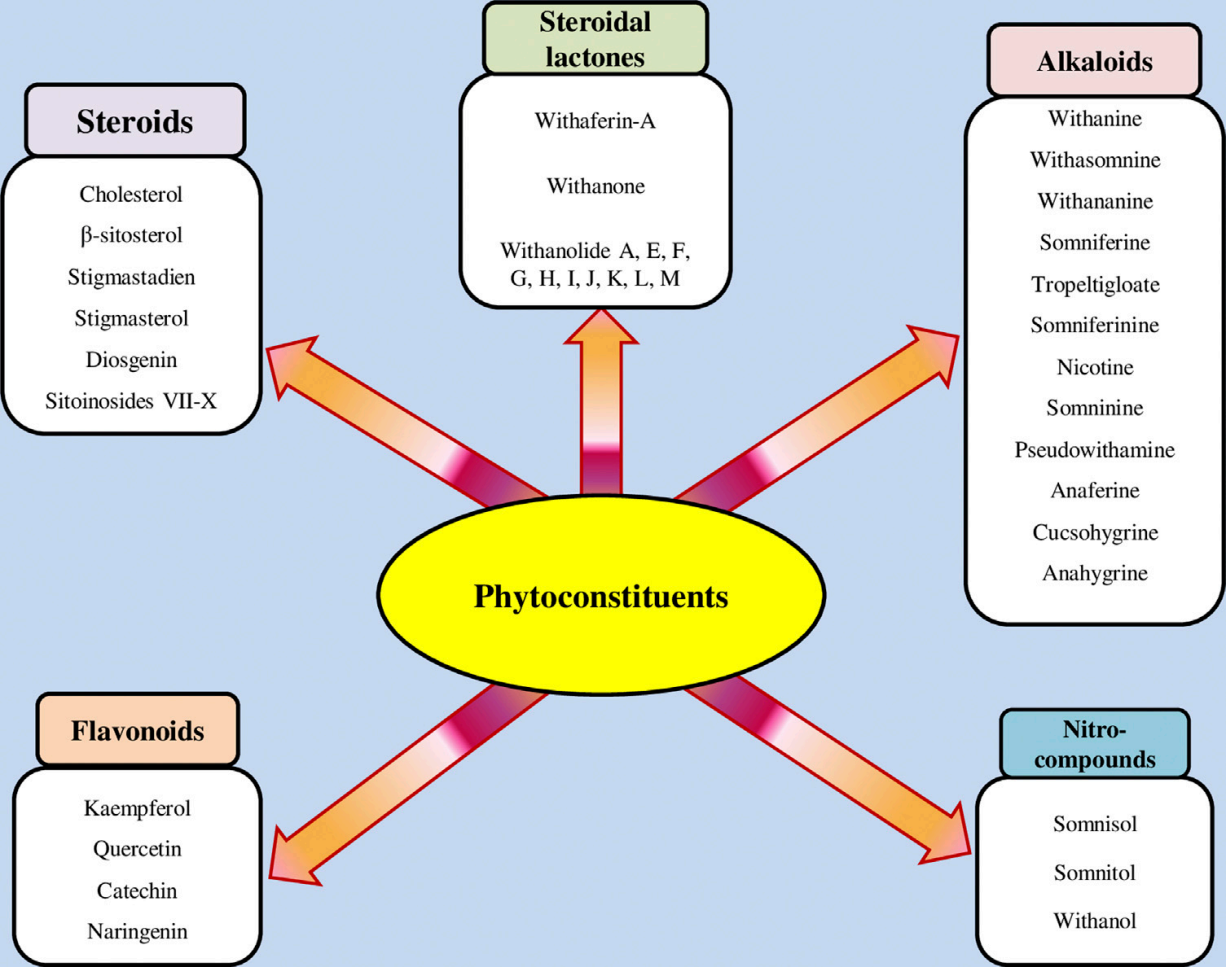
Different compound classes of *W. somnifera*.

The isolation and characterization of secondary plant metabolites are important for the development of new therapeutics to address various health conditions (Nabi et al., 2022). A large number of phytochemicals have been isolated and identified from *W. somnifera* using various chromatographic and spectroscopic analytical techniques such as column chromatography, gas chromatography-mass spectroscopy (GC-MS), liquid chromatography-mass spectroscopy (LC-MS), nuclear magnetic resonance (NMR), and X-Ray diffraction studies (Tong et al., 2011; Paul et al., 2021; Tewari et al., 2022). Various phytochemical studies have revealed the presence of different bioactive constituents from various parts of *W. somnifera* (Figure 1). Several preliminary phytochemical screenings indicated the presence of steroidal lactones, alkaloids, saponin, flavonoids, tannin, starch, phenolic content, carbohydrate, withanolides, sitoindosides, anaferine, anahygrine, *ß*-sitosterol, chlorogenic acid, cysteine, cuscohygrine, pseudotropine, withanine, scopoletin, withananine, somniferinine, somniferiene, tropanol, 14-α-hydroxywithanone, and 6,7β-Epoxywithanon (Saleem et al., 2020). Power and Salway, (1911) isolated phytocompompouds *viz.*, withaniol, somnirol, somnitol, withanic acid, phytosterol, ipuranol, and alkaloids (such as somniferine, somniferinine, withamine, withanmine, pseudowithamine, and withanaminine, etc.) from the alcoholic leaf and root extracts of *W. somnifera*. The first withanolide isolated from *W. somnifera* was Withaferin-A by Lavie et al. (1965). Other withanolides present are Withanolide-A, Withanolide-E, Withanone, etc., (Ali et al., 2015; Saleem et al., 2020). The methanolic leaf extract showed the existence of tisopelletierine, 3α-tigloyloxtropine, cuscohygrine, hentriacontane, visamine, etc., (Afewerky et al., 2021) reducing sugars, ducitol, starch, iron, and some amino acids such as glutamic acid, cysteine, tryptophan, etc., (Alam et al., 2011). In addition, steroids like cholesterol, diosgenin, stigmastadien, sitoinosides VII-X (Bharti et al., 2016) have been reported in the plant. Matsuda et al. (2001) isolated seven new withanosides glycosides *viz.*, withanosides I-VII, and four known compounds such as withaferin A, 5α,20αF (R)-dihydroxy-6α,7α-epoxy-1-oxowitha-2,24-dienolide, physagulin D, and coagulin Q from the methanol root extract of *W. somnifera*. The bioactive compounds and their reported bioactivities are presented in Table 1.

**TABLE 1:** Significant bioactive compounds isolated from *W. somnifera*.

Phytoconstituent	Plant part used	Compound structure	Biological activity	Model used	References
Withaferin-A	Leaf	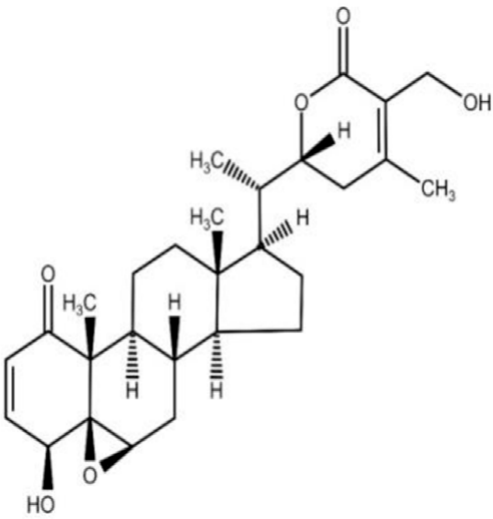	Neuroprotective, cardioprotective	*Invitro* model: C6 glial cell line model	Konar et al. (2011); Dar et al. (2015); Saleem et al. (2020)
				* Invivo* model: Swiss albino mice model	
Withanone	Fruit	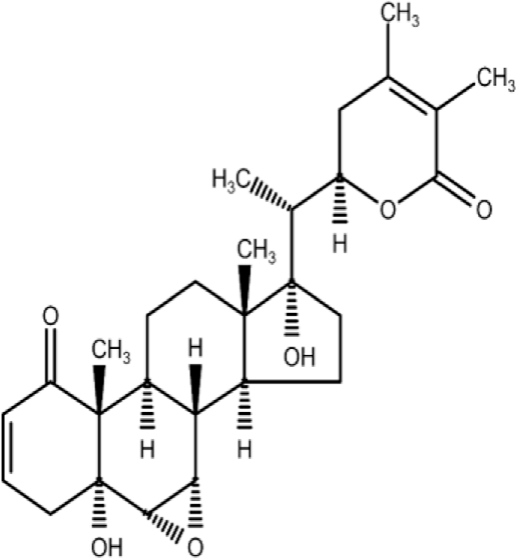	Anti-inflammatory	*Invitro* model: LPS induced bone derived macrophages	Dar et al. (2015); Purushotham et al. (2017); Saleem et al. (2020)
Withanolide-A	Root	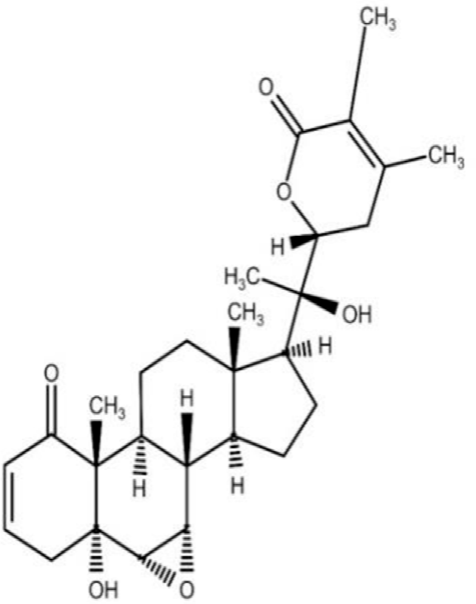	Immunomodulatory	*Invivo* model: BALB/c mice model	Dar et al. (2015); Saleem et al. (2020)
Withanolide-D	Leaf	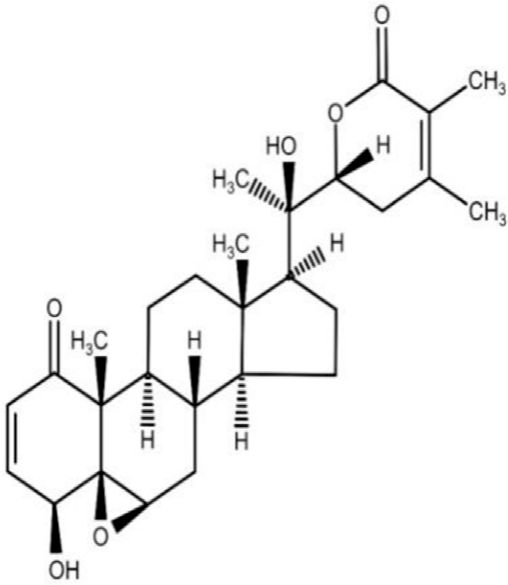	Anti-cancer	*Invivo* model: tumor mice model	Dar et al. (2015); Saleem et al. (2020)
Withanolide-E	Leaf	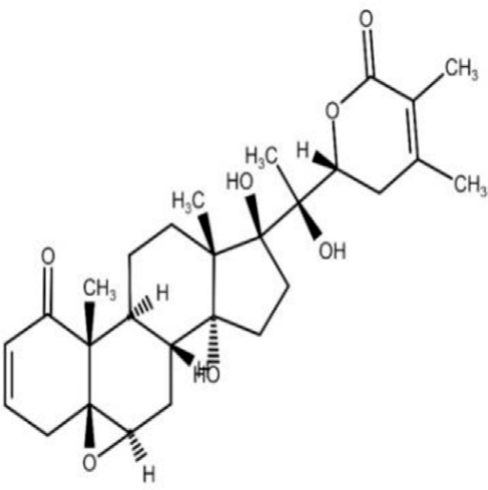	Anti-cancer	*Invitro* model: pancreatic cancer cell lines	Dar et al. (2015); Saleem et al. (2020)
Withanolide-F	Leaf	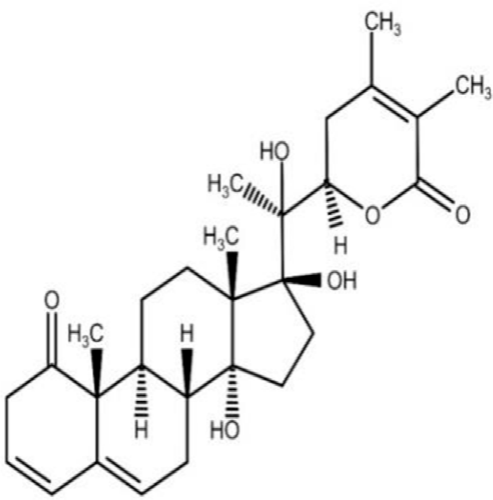	Anti-cancer	*Invitro* model: colon cancer cell lines	Dar et al. (2015); Seth et al. (2016); Saleem et al. (2020)

Note: Chemical structures were drawn using Swiss ADME open-source website.

### Molecular targets of *W. somnifera*

The primary bioactive constituents present in *W. somnifera* are the steroidal lactones known as withanolides (withaferin A and withanolide D), which target different biomolecules in the living systems and are responsible for their diverse pharmacological activities (Paul et al., 2021). Various *invivo* and *invitro* studies have shown that these two compounds target enzymes like kinases, growth factors, transcription factors, receptors, and structural proteins (Sari et al., 2020; Tewari et al., 2022). These have proven therapeutic potential related to the central nervous system (CNS), cardiovascular system, cancer, and inflammatory and metabolic disorders (Dar et al., 2015). The root extract of W. *somnifera* has been reported to possess a potent inhibitory effect on inflammatory markers such as cytokines (IL-2 and IL-8) in mouse models (Khan et al., 2019). Withaferin A and Withanolide D from root extract inhibited the growth of cancer cells and increased their apoptosis by causing up and downregulation of many biomolecules, such as upregulation of caspases-3, etc., which increases apoptosis in cancer cells (Ahmed et al., 2018). Withaferin A suppresses cancer *via* activating tumor suppressor proteins (Sari et al., 2020). They also can act by reducing the expression of estrogen receptors that bind to endogenous estrogen to inhibit the growth of cancer cells (Khazal et al., 2013). The root extract of *W. somnifera* has also been shown to mitigate memory loss in male rats by increasing reduced glutathione (GSH) levels through activation of glutathione biosynthesis in hippocampal cells showing some benefit in Alzheimer’s disease (Baitharu et al., 2014). Different extracts and bioactive compounds of *W. somnifera* have enormous potential to target multiple biomolecules involved in the pathogenesis of various diseases, which are discussed in the following sections.

### Anti-inflammatory/antiarthritic/analgesic activity

**FIGURE 2:**
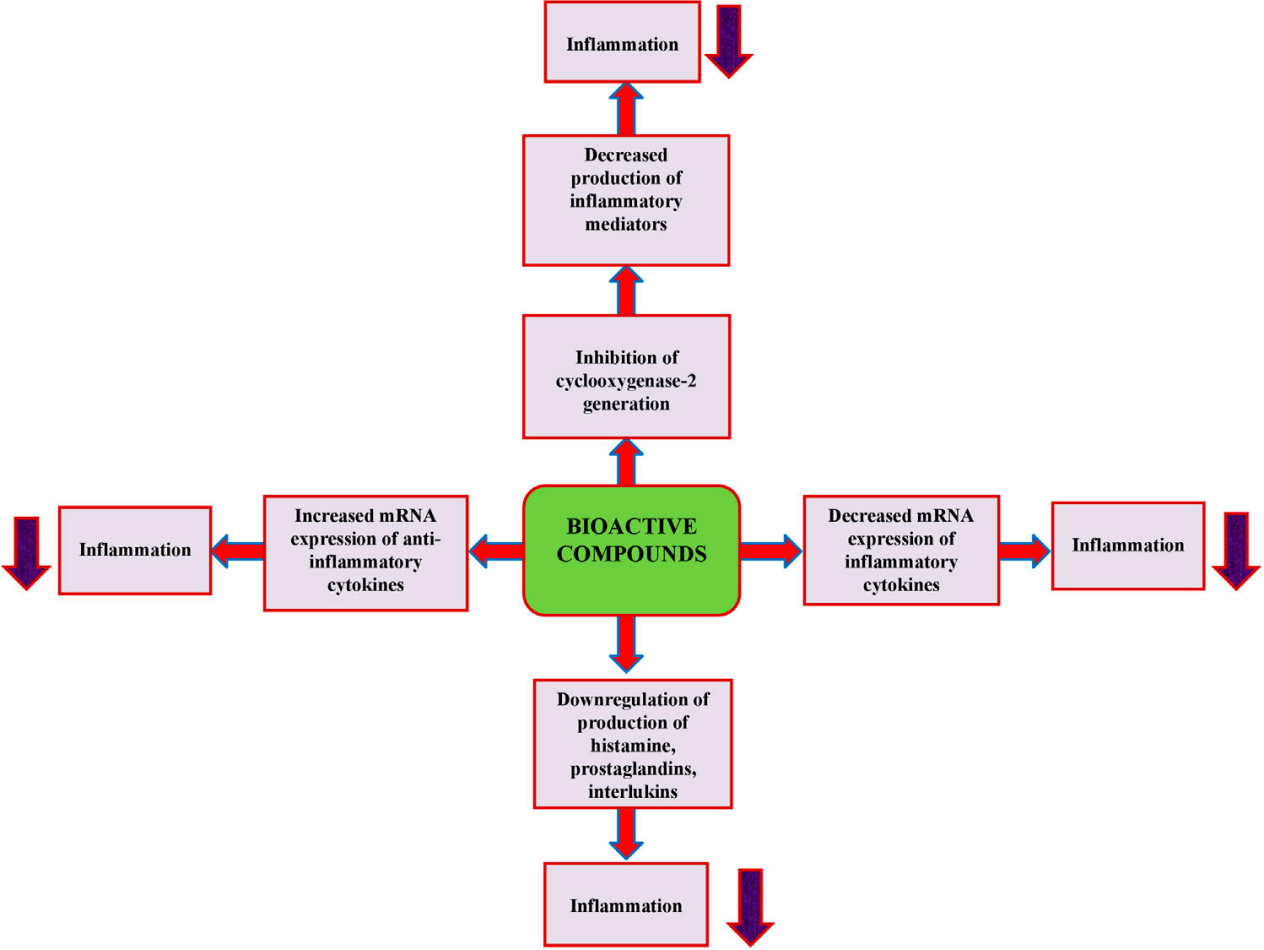
Anti-inflammatory mechanism of bioactive constituents of *W. somnifera*.

*W. somnifera* has been found to exhibit excellent anti-inflammatory activities in several *invitro* and *invivo* models (Paul et al., 2021; Figure 2). *W. somnifera* root extract has shown decreased mRNA expression of inflammatory cytokines such as interleukin 2 (IL-2), interleukin 8 (IL-8), and tumor necrosis factor (TNF), whereas increased the mRNA expression of anti-inflammatory cytokine transforming growth factor (TGF) in a human keratinocyte cell line (HaCaT) (Sikandan et al., 2018; Parihar, 2022b). Furthermore, Sikandan et al. (2018) evaluated the anti-inflammatory activity of the aqueous root extract of *W. somnifera* topically applied to the wounded skin of 7-week-old male C57BL/6J mice for 5 days at a concentration of 10 mg/mL. The results demonstrated inhibition of the pro-inflammatory cytokine TNF-α and increased anti-inflammatory cytokine TGF-β1 mRNA expression. In the wound-healing assay, the extract-treated skin showed a considerable decrease in the wound area and less immune cell aggregation than the control-treated skin. Withaferin-A is effective in treating various inflammatory conditions in diseases such as inflammation associated with arthritis, cystic fibrosis, and inflammatory bowel disease by different mechanisms like inhibiting nuclear factor kappa B (NF-κB) activation, and inhibition of cyclooxygenase-2 (COX-2) generation. Withaferin-A has been shown to increase the expression of an osteoblast-specific transcription factor, which improves osteoblast differentiation and growth in menopausal osteoporosis and bone damage (Khedgikar et al., 2013; Kumar et al., 2015). Withaferin-A has been shown to reduce the cecal ligation and puncture (CLP)-induced endothelial protein C receptor (EPCR) shedding by reducing the expression and activity of tumor necrosis factor-α converting enzyme in mice (Ku et al., 2014; Dar et al., 2015). Both *W. somnifera* root extract and its bioactive compound (withaferin-A), downregulate the production of inflammatory mediators such as histamines, prostaglandins, and interlukins (Gupta and Singh, 2014; Kumar et al., 2015). The aqueous root extract of *W. somnifera* showed a transient chondroprotective effect on damaged human osteoarthritic cartilage by significant and reproducible inhibition of the gelatinase activity of collagenase type-2 enzyme *invitro* models in which explants from osteoarthritis patients were used (Dar et al., 2015). The crude ethanolic extract of *W. somnifera* suppressed lipopolysaccharide (LPS) induced secretion of pro-inflammatory cytokines in synovial fluid mononuclear cells from rheumatoid arthritis patients, possibly by inhibiting nuclear translocation of the transcription factor NF-κB (Dar et al., 2015). *W. somnifera* aqueous root extract decreased the production of pro-inflammatory cytokines mediated *via* inhibition of NF-κB activity in arthritic rats (Khan et al., 2019). By bringing the levels of ROS and metaloproteinase-8 in rats with collagen-induced arthritis back to normal, oral administration of *W. somnifera* aqueous root extract (300 mg/kg) reduced the transcription factors of arthritis in those animals (Khan et al., 2019; Saleem et al., 2020). The methanolic root extract of *W. somnifera* prolonged the morphine-induced analgesia by possibly involving activation of peroxisome proliferator-activated receptor γ in male Sprague rats because the antagonist of this nuclear receptor GW-9662 attenuates the morphine-induced analgesia (de Guglielmo et al., 2014; Orrù et al., 2014).

### Antiparkinson activity

Parkinson’s disease (PD) has been associated with both mitochondrial dysfunction and oxidative stress. Oxidative stress affects dopaminergic neurons, cholinergic receptors, and several other structures implicated in neurodegeneration, triggering a chain of events that includes mitochondrial malfunction and neuroinflammation (Nabi and Tabassum, 2022). Leucine-Rich Repeat Kinase 2 (LRRK2), also known as dardarin (Domingos et al., 2019), is a large protein mutated in patients with familial PD, increased levels of this protein are associated with neuronal toxicity (Pérez-Carrión et al., 2022). It is stabilized by the chaperone heat shock protein 90 (Hsp90) and its co-chaperone Cdc37. The microglial cell line N9 treated with withaferin-A reduced cellular levels of LRRK2 in a concentration and time-dependent manner, disrupting the interaction between Hsp90, Cdc37, and LRRK2, which resulted in LRRK2 instability and downregulation. Furthermore, celastrol (an inhibitor of the Hsp90-Cdc37 complex) reduced LRRK2 levels, while Withaferin-A increased LRRK2 clearance in the presence of celastrol (Narayan et al., 2015). 6-Hydroxy dopamine and 1-methyl-4-phenyl-1,2,3,6 tetrahydropyridine (MPTP) are used to evoke PD symptoms in animal models (Ferreira-Junior et al., 2019). Oral administration of ethanolic root extract of *W. somnifera* at a dose of 300 mg/kg/day improved gripping ability, motor movements, and increased dopamine levels in the striatum of male Wistar rats in the 6-hydroxy dopamine-induced model, as well as maneb and paraquat models, by quenching free radicals and thereby protecting the dopaminergic neurons. Increased levels of antioxidant enzymes such as glutathione peroxidase, catalase, and glutathione reductase were observed, whereas indications of oxidative stress such as lipid peroxidation and nitrite levels were decreased (Prakash et al., 2013). Likewise, Prakash et al. (2014) demonstrated that ethanolic root extract of *W. somnifera* at a dose of 100 mg/kg for 9 weeks provided nigrostriatal dopaminergic neuroprotection against maneb and paraquat-induced Parkinsonism *via* modulation of oxidative stress, significant improvements in canonical PD indicators for instance, impaired locomotor activity, improved pro-apoptotic state *via* reducing Bax and inducing Bcl-2 protein expression, decreased dopamine in the substantia nigra, decreased iNOS expression, and GFAP (a pro-inflammatory marker of astrocyte activation).

### Anti-cancer activity

**FIGURE 3:**
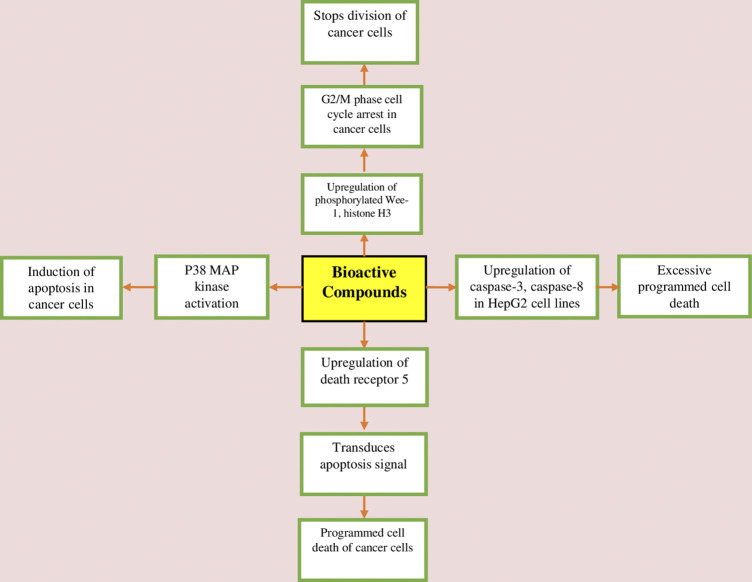
Anticancer mechanism of bioactive constituents of *W. somnifera*.

Cancer is characterized by uncontrolled cell proliferation, and existing chemotherapeutic therapies target various cell signalling pathways and have a direct cytotoxic effect on cells to suppress cancer growth, but withdrawal symptoms limit their use (Amjad et al., 2021).*W. somnifera* has shown a positive safety profile and remarkable anticancer potency in animal models (Palliyaguru et al., 2016). Withaferin-A induces apoptosis in cancer cells *via* mechanisms such as inhibiting the activation of NF-κB by preventing the TNF-induced activation of IκB kinase *ß via* a thioalkylation sensitive redox mechanism, activation of tumor suppresser proteins such as p53 and pRB (Kumar et al., 2015; Sari et al., 2020). Withaferin-A has also been shown to upregulate death receptor-5 and transduces apoptosis signal, which results in programmed cell death of cancer cells (Um et al., 2012; Patel et al., 2013; Kumar et al., 2015). Withaferin-A increases Par-4 induction and p38 MAP kinase activation to induce programmed cell death in cancer cells (Patel et al., 2013; Kumar et al., 2015). On prostate cancer cells, withaferin-A arrests the G2/M phase cell cycle and prevents mitosis by upregulation of phosphorylated Wee-1, phosphorylated histone H3, p21, and aurora B targets (Roy et al., 2013; Kumar et al., 2015). Withaferin-A also possesses its apoptotic action on human colon cancer cells through inhibition of the Notch-1 signalling pathway and down-regulating pro-survival pathways, such as Akt/NF-κB/Bcl-2 in three colon cancer cell lines (HCT-116, SW-480, and SW-620) (Koduru et al., 2010; Kumar et al., 2015; Tewari et al., 2022). It also induces apoptosis in human breast cancer cells through mechanisms such as inhibition of cell migration/invasion through downregulation of signal transducer and activator of transcription (STAT3) activity (Patel et al., 2013; Kumar et al., 2015; Tewari et al., 2022). Withaferin-A increases cytotoxicity in HepG2 (hepatocellular carcinoma) through upregulation of caspase-3, caspase-8, and caspase-9, which resulted in excessive programmed cell death (Ahmed et al., 2018; Tewari et al., 2022). Withaferin-A decreases cell survival in B cell lymphoma cell line by downregulation of heat shock protein 90 (HSP90), as this protein stabilizes several proteins required for tumor growth (McKenna et al., 2015; Tewari et al., 2022). *W. somnifera* root extract reduced cell viability and G2/M phase cell cycle arrest in a prostate cell line (PC3) by downregulation of transcription factors, which resulted in decreased biosynthesis of IL-8 and COX-2 enzymes (Setty Balakrishnan et al., 2017; Tewari et al., 2022). Hahm and Singh (2013) used 2.5 uM and 5 uM dosages of Withaferin-A on MDA-MB-231 and MCF-7 human breast cancer cells. The study revealed the molecular intricacies governing Withaferin-A-mediated IAP dysregulation. Survivin and cIAP-2 mRNA levels were reduced by Withaferin-A treatment, although XIAP mRNA levels were only minimally affected. The stability of cIAP-2, XIAP, or Survivin mRNA was not altered, following treatment with withaferin-A, at least in the MDA-MB231 cell line. Khazal et al. (2013) used mammary carcinogen methylnitrosourea (MNU) to induce breast cancer in female Sprague-Dawley rats and reported that treatment with *W. somnifera* root extract at a dose of 150 mg/kg bw for 155 days decreased the number of tumours by 23% when compared to the control group. Withaferin-A *via* blocking IL-6-induced STAT3 phosphorylation caused concentration-dependent apoptotic cell death in Caki human renal cells (Um et al., 2012; Tewari et al., 2022). In another study, withaferin-A induced apoptosis in renal carcinoma cells mediated by endoplasmic reticulum stress, which upregulated the transcription factor CCAAT/enhancer-binding protein homologous protein (CHOP) (Choi et al., 2011). The anti-cancer potential of bioconstituents of *W. somniferais* presented in Figure 3.

### Neuroprotective activity

**FIGURE 4:**
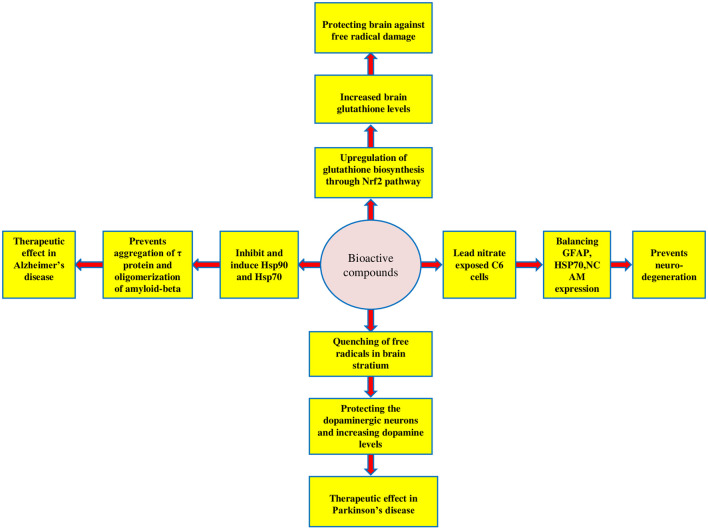
Neuroprotective mechanism of bioactive constituents of *W. somnifera*.

*W. somnifera* has proven to perform a neuroprotective role in various preclinical and clinical studies (Pandey et al., 2018; Paul et al., 2021; Figure 4). *W. somnifera* root extract downregulated NO production by interacting with nNOS, which inhibited the stress-induced NADPH-diaphorase activation by suppressing corticosterone release and activating choline acetyltransferase which increased the serotonin level in the hippocampus to inhibit NADPH-d in adult Swiss albino mice exposed to resistant stress (Bhatnagar et al., 2009). Another study performed on adult Swiss albino mice induced oxidative stress *via* ROS generation, reducing the antioxidant cell defence by depleting glutathione when exposed to lead nitrite. The administration of hydroalcoholic root extract of *W. somnifera* at doses 200 mg/kg/day and 500 mg/kg/day for 6 weeks increased the brain antioxidant enzymes like superoxide dismutase, choloamphenicol acetyltransferase, glutathione s-transferase by scavenging ROS, which contributes to the protective effect of *W. somnifera* (Sharma et al., 2011). The neuroprotective potential of W. somnifera has also been proved by exposing C6 cells to lead nitrite by balancing glial fibrillary acidic protein (GFAP) expression, heat shock protein (HSP70), and neural cell adhesion molecule (NCAM) expression, the unbalance of the same results in neurodegeneration (Kumar et al., 2014). Konar et al. (2011) treated neuronal and glial cell lines IMR32 and C6 with scopolamine, which resulted in the downregulation of brain-derived neurotrophic factor (BDNF) and glial fibrillary acidic protein (GFAP). Both are significant for neuronal and glial cells normal growth and upregulation of oxidative stress ROS markers. Withanone upregulated the production of the former and downregulation of the latter. Similarly, Swiss albino mice treated with *W. somnifera* leaf extract at a dosage of 100–300 mg/kg/day exhibited an increase in brain-derived neurotrophic factor (BDNF) and glial fibrillary acidic protein (GFAP), both of which are downregulated when treated with scopolamine alone, indicating brain injury. Kataria et al. (2012) evaluated the neuroprotective effect of the aqueous leaf extract of *W. somnifera* using rat glioma (C6) and human neuroblastoma (IMR0-32) cell lines. The MTT assay was used to assess cell viability and immunocytofluoroscence and Western blot techniques were used to check levels of HSP70. The results demonstrated that the extract protected the retinoic acid, differentiated rat glioma, and human neuroblastoma cells against glutamate-induced toxicity characterized by neuronal cell death and an increase in stress protein HSP70. Baitharu et al. (2014) exposed male Sprague Dawley rats to hypoxia at high altitudes (25,000 ft), which resulted in hippocampal neurodegeneration owing to the formation of free radicals due to low oxygen levels and weakened the antioxidant enzyme system. Administration of Withanolide A at a dosage of 10 μmol/kg BW before 21 days of pre-exposure and during 7 days of exposure to hypoxia increased glutathione levels in neuronal cells by up-regulating enzymes for glutathione biosynthesis and gamma-glutamyl cysteinyl ligase *via* the Nrf2 pathway in a corticosterone dependent manner.

### Anti-epileptic activity

*W. somnifera* and its bioactive constituents such as withanolides investigated *via* various in-vitro and *in-vivo* models proved to be effective in reducing epileptic seizures through many mechanisms (Kumar et al., 2015; Anju et al., 2018). *W. somnifera* extracts and withanolides have been shown to increase the seizure threshold in the brain in pentylenetetrazol-induced seizures with co-administration of a sub-protective dose of GABA or diazepam and the mechanism involved was GABAergic modulation (Kumar et al., 2015). Oral administration of *W. somnifera* root extract and withanolide-A at doses of 100 mg/kg/day and 10 mol/kg/day for 15 days restored spatial memory deficit in male Wistar rats with epilepsy induced by pilocarpine *via* inhibiting oxidative stress-induced alteration in glutamergic transmission in the brain by reducing N-methyl-D-aspartate (NMDA) receptor expression. Furthermore, in a pilocarpine-induced epilepsy model, suppressing the -Amino-3-hydroxy-5-methyl-4-isoxazolepropionic acid receptor (AMPA receptor) expression improved motor learning (Soman et al., 2012). Similarly, Anju et al. (2018) investigated *W. somnifera* root extract and Withanolide-A for 15 days in male Wistar rats at dosages of 100 mg/kg/day and 10 mol/kg/day for inhibiting elevated muscarinic receptor activation in temporal lobe epilepsy, which causes oxidative stress and disrupted cell signalling. The treatment significantly restored the impaired muscarinic receptor activation and oxidative stress and regulated cellular signalling, resulting in a lower prevalence of seizures as compared to the control group.

### Anti-Alzheimer activity

*W. somnifera* root extract and its active principles have shown promising results in the treatment of Alzheimer’s diseases in many *invivo* and *invitro* studies by altering different pathological processes like accumulation of amyloid beta plaques in the brain, increased muscarinic receptor binding affinity, etc. (Jayaprakasam et al., 2010; Grover et al., 2012; Barua et al., 2020; Das et al., 2021). The root extract has been shown to block Aβ production, decreasing apoptotic cell death of neurons through the migration of nuclear factor erythroid 2-related factor 2 (Nrf2) to the nucleus (Farooqui et al., 2018; Das et al., 2021). It is a transcription factor that regulates the activity of antioxidant enzymes and protects the cells against oxidative damage (Saha et al., 2020). The transcription factor increases the expression of the neuroprotective enzyme heme oxygenase-1 (Farooqui et al., 2018; Das et al., 2021). Withaferin-A has been shown to inhibit the heat shock protein 90 (Hsp90) and induce heat shock protein 70 & 27 (Hsp27 & Hsp27), whereas the former causes aggregation of τ protein, which is the hallmark of Alzheimer’s disease, the latter has a protective role because these inhibit the oligomerization of amyloid-beta in mouse and *drosophila* larval model of Alzheimer’s disease (Sinadinos et al., 2013; Blair et al., 2014; Das et al., 2021). The thioflavin T fluorescence assay revealed that withaferin-A reduced the production of Aβ, possibly by increasing *a*-secretase expression and decreasing *ß*-secretase expression. Whereas *a*-secretase prevents the formation of Aβ by cleaving the amyloid precursor protein (APP) within the domain, the *ß* and *?* secretase act through sequential cleavage to produce Aβ. It also increased insulin-degrading enzyme (IDE) production, which causes the degradation of Aβ (Kuboyama et al., 2014; Fan et al., 2020; Das et al., 2021). In an *invitro* study, it has been found to inhibit AChE obtained from electric eels and enhance choline acetyltransferase (ChAT) levels in rats (Behl et al., 2020). These two actions of withaferin-A could lead to increased cholinergic transmission in certain areas of the brain, like the basal ganglia and cerebral cortex, which can lead to improved cognitive function by enhancing the binding of acetylcholine to muscarinic M1 receptor. Also, it has been reported that there was no effect on GABAA, NMDA, glutamate, and benzodiazepine receptors limiting the side effects (Das et al., 2021). In Alzheimer’s disease, the NF-κB pathway blocks the phagocytosis of Aβ fibrils, which leads to the accumulation of Aβ fibrils and neuroinflammation in the brain (Heneka et al., 2013). Withaferin-A has been shown to inhibit the activation of NF-κB by stopping phosphorylation of NF-κB by inhibiting stimulation of IκB kinase. It also inhibited the activation of NF-κB by attacking the catalytic site of IκB kinase and preventing neuroinflammation (Rahman et al., 2020).

### Hepatoprotective activity

Several studies have shown the hepatoprotective potential of *W. somnifera* and its bioactive constituents (Malik et al., 2013; Devkar et al., 2016; Palliyaguru et al., 2016; Sayed et al., 2019; Saleem et al., 2020). Withaferin-A has been reported to diminish the D-galactosamine/lipopolysaccharide-induced acute liver failure in wild type mice by inhibiting the activation of macrophages. The compound improved GalN/LPS-induced hepatotoxicity by targeting macrophage partially dependent on NLRP3 antagonism, while largely independent of NRF2 signaling, autophagy induction, and hepatic AMPKα1 and IκκB (Xia et al., 2021; Xia et al., 2022). Malik et al. (2013) studied hepatoprotective activity of aqueous root extract of *W. somnifera* at a dose of 500 mg/kg bw for 28 days against paracetamol (500 mg/kg bw) induced hepatotoxcity in female Swiss albino mice. The study revealed significant improvement in liver marker enzymes such as AST, ALP, ALT, bilirubin and increased GSH levels compared to control (0.9% NaCl). In another study, Withaferin-A has shown to protect the liver against bromobenzene-induced liver injury in mice by increasing the levels of mitochondrial enzymes, which act as antioxidants by balancing the expression of Bax/Bcl-2 in the liver (Vedi et al., 2014; Vedi and Sabina, 2016). It has also been shown to inhibit the EMT (epithelial-mesenchymal transition) process, which plays a central role in liver fibrosis by inhibiting the expression of some enzymes like metallopeptidase inhibitor 1 (TIMP1), lysyl oxidase homolog 2 (LOXL2), matrix metalloproteinase-2 (MMP2) which enhances the expression of cadherin-1 (CDH1) leading to the reversal of EMT (Zhao et al., 2016; Sayed et al., 2019; Xia et al., 2022). Both withaferin-A and withanone decreased the synthesis of inflammatory cytokines *viz.*, TNFa and IL6 in LPS-induced bone-derived macrophages (Purushotham et al., 2017). Further, Withaferin-A inhibited the mitogen-activated protein kinases, including ERK, JNK, and NFkB activation, whereas withanone only regulated the ERK and JNK signalling pathways. All these kinases and pathways play a significant role in systemic inflammation, including liver (Xia et al., 2022).

### Cardioprotective activity

*W. somnifera* has cardioprotective and cardiotonic properties and is used traditionally for cardiovascular diseases (John, 2014). Various studies on the plant and its bioactive compounds using animal models have proved cardioprotective and cardiotonic effects (Paul et al., 2021). Both *W. somnifera* extract and withaferin-A have shown cardioprotective effects in Wistar rats and mice at doses of 40 mg/kg and 1 mg/kg, respectively. In LAD coronary ligation method and reperfusion-induced myocardial injury by decreasing the apoptotic cell death by up-regulating BCl-2 (anti-apoptotic protein) and down-regulating the Bax (pro-apoptotic protein) and thereby reducing the infarct size in the myocardium (Mohanty et al., 2008). A study by Reuland et al. (2013) on cultured HL-1 cardiomyocytes using an herbal formulation containing *W. somnifera* (0–100 μg/mL) reduced the oxidative damage caused by doxorubicin to these cells by activating transcription factor Nrf-2, which stimulates phase-2 detoxification enzymes which act as antioxidants by scavenging free radicals (Paul et al., 2021). Withaferin-A showed anti-platelet and profibrinolytic effects in post-myocardial infarction in normal human plasma by measuring activated partial thromboplastin-time (aPTT) and prothrombin time (PT). Also, TNF-α stimulated human umbilical vein endothelial cells (HUVECs) and inhibited the synthesis of plasminogen activator inhibitor type 1 (PAI-1) with no direct effect on tissue plasminogen activator (tPA), thereby reducing the PAI-1/t-PA ratio which can exert the fibrinolytic effects (Ku et al., 2014; Paul et al., 2021). Mohanty et al. (2004) evaluated the cardioprotective potential of hydro-alcoholic extract of *W. somnifera* at doses of 25, 50, and 100 mg/kg for 28 days in Wistar albino male rats. A significant decrease in glutathione (*p* < 0.05), activities of superoxide dismutase, catalase, creatinine phosphokinase, lactate dehydrogenase (*p* < 0.01), and an increase in lipid peroxidation marker malonyldialdehyde level (*p* < 0.01) was observed in the hearts of isoproterenol control group rats. The histopathological analysis showed myocardial damage. The increased endogenous antioxidants, maintenance of the myocardial antioxidant status, and significant restoration of altered haemodynamic parameters may contribute to its cardioprotective activity. Guo et al. (2019) evaluated cardioprotective potential at two different doses of withaferin-A in wild-type and AMP-activated protein kinase domain negative (AMPK-DN) transgenic mice. A low dose of 1 mg/kg exerted a cardioprotective effect *via* up-regulating the AMP-activated protein kinase level and decreased the activation of caspase 9 in wild mice, which led to improved cardiac function and reduced infarct size by inhibiting the apoptotic effect of caspase 9, whereas, this protective effect was absent in transgenic mice.

### Cognition enhancing activity

Several pre-clinical and clinical studies have shown that *W. somnifera* enhances the cognitive function in individuals with neurodegenerative disorders, anxiety induced cognitive dysfunction, etc. (Dar and Muzamil, 2020; Ng et al., 2020; Zahiruddin et al., 2020). A dosage of 100 mg/kg/day of *W. somnifera* root extract indicated protection against Propoxur-induced memory loss and reductions in brain and blood cholinesterase activity in male Wistar rats (Yadav et al., 2010). In a study involving Swiss albino mice, 21-day oral treatment of *W. somnifera* root extract at a dose of 100 mg/kg/day was able to protect the brain against cognitive dysfunction induced by bisphenol and exerted this effect *via* direct scavenging of ROS and by modulating the activity of antioxidant enzymes like catalase and SOD. It was also able to upregulate the NMDA receptor activity, which was downregulated by the bisphenol, the downregulation of the NMDA receptor plays a crucial role in cognitive impairment (Birla et al., 2019). Reduced acetylcholinesterase activity has been implicated in memory loss and cognitive dysfunction.

### Antiviral activity against SARS-CoV-2

Various *insilico* studies have shown that bioactive compounds present in the plant have been shown to target a few enzymes and the main spike protein of the virus *via* which it attaches with the host ACE2 receptor and enters the cell (Parihar, 2022a). Withanoside and Somniferine have been shown by molecular docking and dynamic stimulation to exhibit a high binding affinity for SARS-CoV-2 main protease enzyme (Mpro) with high binding affinity and have therapeutic potential against COVID-19 (Shree et al., 2022). Withanoside-V and Withanoside-X by docking studies have been shown to possess a strong binding affinity for viral S-glycoprotein which is responsible for the attachment to human ACE2 receptor, highlighting further therapeutic potential against the disease (Chikhale et al., 2021). However, more elaborate *invitro* and *invivo* studies are needed to investigate the extracts and bioactive compounds of *W. somnifera* and their mode of action against the SARS-CoV-2 virus.

### Pharmacokinetics of *W. somnifera*

Different studies have reported the pharmacokinetic parameters of *W. somnifera* root extracts and the bioactive compounds present in them. Gupta et al. (2022) evaluated the pharmacokinetic parameters of Withaferin-A in female BALB/c mice using a validated LC-MS/MS method by giving a single dose of Withaferin-A orally to one set of mice and i. v to another group. Maximum plasma concentration (Cmax) was found to be 3,996.90 ± 557.6 ng/mL i. v. and 141.7 ± 16.8 ng/mL orally. Tmax (Time taken to achieve maximum plasma concentration was found to be 0.5 h when the dose was given orally. The oral bioavailability was found to be 1.8%. *Insilico* studies by Dai et al. (2019) suggested that the low bioavailability may be due to the extensive first pass metabolism by liver enzymes. Patil et al. (2013) carried out a study on the pharmacokinetic parameters of Withaferin-A and Withanolide A and evaluated the oral administration of an aqueous extract of *W. somnifera* at a dose of 1,000 mg/kg of body weight to Swiss albino mice *via* HPLC-MS/MS method. Maximum plasma concentration of Withaferin-A and Withanolide A was found to be 16.69 ± 4.02 ng/mL and 26.59 ± 4.47 ng/mL at Tmax of 10 and 20 min, respectively, suggesting rapid absorption of these bioactive compounds.

### Clinical trials of *W. somnifera*

With its widespread use all over the world, it has become necessary to scientifically evaluate all the claimed uses of the root extracts of the herb and other formulations used in the traditional systems of medicine. Many clinical trials have been done to evaluate the efficacy of the herb in relation to human consumption (Tandon and Yadav, 2020; Table 2). In a randomized, double-blind controlled study standardized root extract of herb in a dose of 1 gm/day for 12 weeks showed a reduction in positive and negative symptoms in schizophrenia as compared to a placebo (Chengappa et al., 2018). In another study anxiety and depression associated with schizophrenia were improved with 1 gm/day of standardized extract of the root compared to a placebo in the 12-week study on 66 patients (Gannon et al., 2019). The aqueous root extract of 600 mg/day for 8 weeks in a double-blind randomized controlled trial in a hospital setting resulted in normalization of TSH, T3, and T4 in sub-clinical thyroid patients against a placebo (Sharma et al., 2018). In a triple-blind randomized control trial in 100 patients with idiopathic infertility and oligospermia administration of capsules containing dried root powder of the herb 5 gm/day was given to one group of patients and the other group received pentoxifylline 800 mg/day for 90 days, both groups showed a significant increase in sperm count and motility and no adverse effect was reported except nausea and epigastric pain in 1 patient, so *W. somnifera* can be an alternative to pentoxifylline in treating male infertility with good safety profile (Nasimi Doost Azgomi et al., 2018). In a randomized double-blind controlled study involving 66 patients with diabetes mellitus already on metformin therapy received either 250 mg or 500 mg of the aqueous root extract or placebo as an add-on therapy to metformin for 12 weeks. *W. somnifera* group showed significant improvement in HbA1c levels, total cholesterol, triglycerides, and LDL, biomarkers of oxidative stress and systemic inflammation were also improved compared to a placebo (Usharani et al., 2014). In another clinical trial, the efficacy of *W. somnifera* root extract in chronic stress in 64 patients was done against a placebo for a period of 60 days. In this double-blind randomized controlled placebo trial standardized root extract was given in a dose of 300 mg twice daily in the form of a capsule to one group of patients and a placebo was given to another group. The herb-treated group showed a significant reduction in perceived stress score of 44% reduction as compared to only 5.5% in the placebo group, serum cortisol levels were also reduced as compared to the placebo (Chandrasekhar et al., 2012).

**TABLE 2:** Efficacy and adverse effects of root extracts of *W. somnifera* in some clinical trials.

Type of formulation and dose	Pathophysiological condition	Efficacy	Adverse effects	References
Aqueous root extract (300 mg twice daily in capsule dosage form	Chronic stress	Reduction in cortisol level and improvement in PSS	No effect on vital signs of the body and mild hyperacidity in 2 subjects out of 52	Choudhary et al. (2017a)
Aqueous root extract (300 mg twice daily in capsule dosage form)	Insomnia	Improvement in sleep onset latency and total sleep time	No adverse effect reported by any subject	Langade et al. (2019)
Branded root extract (1 gm/day in capsule dosage form	Schizophrenia	Significant reduction in positive and negative symptoms	Mild to moderate GI effects, headache, dry mouthetc.	Chengappa et al. (2018)
100% aqueous root extract (300 mg twice daily in capsule dosage form)	Cognitive impairment	Improvement in memory as seen on different memory tests and scales	No adverse effects reported	Choudhary et al. (2017b)
Aqueous root extract (600 mg/day in capsule form	Sub-clinical hypothyroidism	Normalization of serum TSH, T3, and T4	Cough and headache in 1 subject	Sharma et al. (2018)
Dried root powder (5 gm/day in capsule form)	Idiopathic infertility and oligospermia	Improvement in sperm count, motility, and morphology	Nausea and stomach pain in 1 subject	Nasimi Doost Azgomi et al. (2018)

### Future prospects

*W. somnifera* is one of the most extensively used medicinal plants in the traditional Indian System of Medicine for a plethora of diseases, and the claims for its efficacy as a multifaceted therapeutic agent to ameliorate a broad spectrum of clinical illnesses are unanimously positive. Various *W. somnifera* extracts have been widely analyzed in pre-clinical *invitro* and *invivo* models, suggesting potential molecular targets of *W. somnifera* as well as its efficacy in alleviating a wide range of human ailments. Several *invitro* and *invivo* studies such as anti-inflammatory, antiarthritic, analgesic, antiparkinson, anticancer, anti-Alzheimer, anti-epileptic, neuroprotective, cardioprotective, hepatoprotective, antiviral, etc., are promising. However, there are inadequate clinically proven studies to validate the broad therapeutic use of its active principles, rather there is a gap in results pertaining to various human diseases that could be addressed by developing a well-regulated larger group of studies to investigate the effect of this promising therapeutic candidate.

### Limitations

*W. somnifera* has excellent therapeutic potential, but all of the studies that have evaluated its potential have been pre-clinical. Thus, limiting its use in allopathic medicine and thereby preventing it to extrapolate to human settings unless undertaken clinically on a broad scale involving a large number of participants. Therefore, extensive clinical investigations are required to uncover the constraints in pharmacodynamics.

## Conclusion

The bioactive compounds present in*W. somnifera*, such as withanolides possess potential therapeutic agents against many disorders of CNS, cyclical vomiting syndrome (CVS), inflammatory disorders, and liver diseases as they target various biomolecules in living systems which play a major role in these disorders and have proven benefit in animal models of disease. Withanolides such as withaferin-A target multiple pathways of inflammation, cancer, neurodegenerative disorders, etc. However, more clinical trials need to be performed against different forms of cancer and other copious health conditions. The low incidence of side effects of *W. somnifera* makes it the safer choice for its use against various human ailments, but further studies on human subjects are necessary. The proven safety profile of this plant species makes the bioactive compounds potential candidates for clinical trials.

## Glossary

AMPA receptor: α-Amino-3-hydroxy-5-methyl-4-isoxazolepropionic acid receptor
Aptt: activated partial thromboplastin-time
BDNF: brain derived neurotrophic factor
CHAT: choline acetyltransferase
CHOP: CCAAT/enhancer-binding protein homologous protein
CNS: central nervous system
COX-2: cyclooxygenase-2
CVS: cyclical vomiting syndrome
DNA: deoxyribonucleic acid
EPCR: endothelial protein C receptor
GC-MS: gas chromatography-Mass spectroscopy
GFAP: glial fibrillary acidic protein
GSH: glutathione
HepG2: hepatocellular carcinoma
Hsp90: heat shock protein 90
HUVECs: human umbilical vein endothelial cells
IDE: insulin degrading enzyme
IL-2: interleukin 2
IL-8: interleukin 8
LC-MS: liquid chromatography-mass spectroscopy
LPS: lipopolysaccharide
LRRK2: leucine-rich repeat kinase 2
Mpro: main protease
MPTP: 1-methyl-4-phenyl-1,2,3,6 tetrahydropyridine
mRNA: messenger ribonucleic acid
NF-κB: nuclear factor kappa-B
NHEJ: non-homologous end joining
NMDA: N-methyl-D-aspartate
NMR: nuclear magnetic resonance
PAI-1: plasminogen activator inhibitor type 1
PD: parkinson’s disease
PT: prothrombin time
ROS: reactive oxygen species
SARS-CoV-2: severe acute respiratory syndrome coronavirus 2
STAT3: signal transducer and activator of transcription
TGF: transforming growth factor
TNF: tumor necrosis factor
TPA: tissue plasminogen activator
